# On a new crested pterodactyloid from the Early Cretaceous of the Iberian Peninsula and the radiation of the clade Anhangueria

**DOI:** 10.1038/s41598-019-41280-4

**Published:** 2019-03-20

**Authors:** Borja Holgado, Rodrigo V. Pêgas, José Ignacio Canudo, Josep Fortuny, Taissa Rodrigues, Julio Company, Alexander W. A. Kellner

**Affiliations:** 1 Laboratory of Systematics and Taphonomy of Fossil Vertebrates, Departmento de Geologia e Paleontologia, Museu Nacional/UFRJ, Rio de Janeiro, Brazil; 2 Institut Català de Paleontologia Miquel Crusafont, Universitat Autònoma de Barcelona, Cerdanyola del Vallès, Catalonia Spain; 3 Grupo Aragosaurus-IUCA, Área de Paleontología, Facultad de Ciencias, Universidad de Zaragoza, Zaragoza, Spain; 4 Museo de Ciencias Naturales de la Universidad de Zaragoza, Zaragoza, Spain; 5 Laboratório de Paleontologia, Universidade Federal do Espírito Santo, Vitória, Brazil; 6 Departamento de Ingeniería del Terreno, Universidad Politécnica de Valencia, Valencia, Spain

## Abstract

The pterosaur record from the Iberian Peninsula is mostly scarce and undefined, but in the last few years some new taxa have been described from different Lower Cretaceous sites of Spain. Here we describe a new genus and species of toothed pterodactyloid pterosaur from the Barremian of the Iberian Peninsula, *Iberodactylus andreui* gen. et sp. nov., that shows a close and rather unexpected relationship with *Hamipterus tianshanensis* from China. A review of the phylogenetic relationships of the Anhangueria reveals a new family of pterodactyloid pterosaurs, the Hamipteridae fam. nov. being recovered as sister-group of the Anhangueridae. This latter clade can be in turn divided into the new clades Anhanguerinae and Coloborhynchinae. The close relationships of *Iberodactylus* and *Hamipterus* shows an interesting palaeobiogeographical correlation between the Chinese and Iberian pterosaur faunas during the Barremian (Lower Cretaceous). The discovery of *Iberodactylus* strongly suggests that the clade Anhangueria has clear ancestral ties in eastern Laurasia.

## Introduction

The first vertebrates to develop powered flight were the pterosaurs, a lineage of archosaurs that occupied the Mesozoic skies all over the world for over 160 Ma^1–5^. They evolved their anatomy and proportions into well over a hundred species, achieving the largest sizes and wingspans of all flying animals^5^. Notwithstanding their distribution, their record is rather patchy, with most occurrences limited to fragmentary remains that in several cases were only briefly reported in the literature^6^. The pterosaur record from the Iberian Peninsula is mostly scarce and undefined^7^, but in the last few years some new taxa have been described from different Lower Cretaceous sites of Spain^8,9^.

Here, we describe a new pterosaur species from the Barremian of the Blesa Formation, *Iberodactylus andreui* gen. et sp. nov., represented by a partial rostrum including a partial premaxillary crest and six pairs of tooth sockets. Our phylogenetic analysis supports a sister-group relationship between the new species and the Chinese *Hamipterus tianshanensis*^10,11^, joined here in the new clade Hamipteridae.

**Figure 1:**
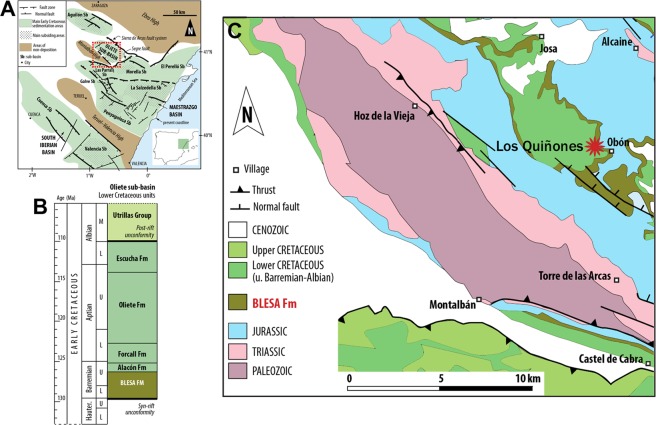
Geographical and geological location of the Los Quiñones site in the Blesa Formation. (**A**) Geological map of the Iberian Peninsula; (**B**) Location of the paleogeographical sub-basins within the Maestrazgo Basin; and (**C**) location of the Los Quiñones site close to the village of Obón (modified from^14^).

The specimen was recovered from the Los Quiñones site at the end of the 1980s by the local collector Mr. Javier Andreu, and was preliminary reported as an ornithocheroid pterosaur^12^. Los Quiñones is close to the village of Obón (Teruel, Spain), located in the northeast of the Iberian Peninsula (Fig. 1), and placed within the upper part of the Blesa Formation, which is considered as Barremian in age^13,14^. The Blesa Formation is part of the syn-rift sedimentation in the Iberian Basin during the Early Cretaceous, and has been recently divided in 3 genetic stratigraphic sequences: Lower, Middle, and Upper Blesa sequences^14^. The specimen studied in this work was found in a limestone layer from the lower part of the Middle Blesa sequence, known as Morenillo member. Even though most of the Blesa Formation is deposited in a continental environment with abundant terrestrial tetrapod remains, the Middle Blesa sequence was deposited in a coastal environment rich in marine fossils as mollusc bivalves, but where isolated remains of actinopterygians, chondrichthyans, chelonian plates, teeth and cranial and postcranial elements of crocodylomorphs are also found^13,15^, as well as teeth and vertebral centra belonging to plesiosaurs^16^.

Furthermore, we present a new proposal for the interrelationships of the Anhangueria. As defined by Rodrigues and Kellner^17^, the Anhangueria constitutes a clade comprising all taxa closer to *Anhanguera* than to *Cimoliopterus*. On the basis of previous studies about crested pterodactyloids^9,17–20^, we performed a phylogenetic analysis focusing on derived pterodactyloids with particular emphasis on anhanguerians^17^.

## Results

### Systematic Palaeontology

Pterosauria Kaup, 1834.

Pterodactyloidea Plieninger, 1901.

Ornithocheiroidea Seeley, 1870 *sensu* Kellner^18^.

Pteranodontoidea Marsh, 1876 *sensu* Kellner^18^.

Lanceodontia Andres *et al*.^21^.

Ornithocheirae Seeley, 1870 *sensu* Andres *et al*.^21^.

Anhangueria Rodrigues & Kellner, 2013.

Hamipteridae fam. nov.

#### Branch-based definition

The most inclusive clade containing *Hamipterus tianshanensis*, but not *Ludodactylus sibbicki*, *Coloborhynchus clavirostris*, and *Anhanguera blittersdorffi*.

#### Diagnosis

Crested anhanguerian pterodactyloids with the following synapomorphies: well-defined parallel and forward curved striae and sulci on the anterior region of the premaxillary crest, and an anterior rounded expansion of the anterior margin of the premaxillary crest.

#### Included species

*Hamipterus tianshanensis* and *Iberodactylus andreui* gen. et sp. nov.

*Iberodactylus andreui* gen. et sp. nov.

#### Etymology

From the Iberian Peninsula and the Iberian System, where the specimen was recovered, and ‘dactylos’ (δάκτυλος), finger (ancient Greek), a common suffix in pterosaur names; in honour of Mr. Javier Andreu, a local collector who found the fossil.

#### Holotype

Museo de Ciencias Naturales de la Universidad de Zaragoza (MPZ, Zaragoza, Spain) MPZ-2014/1; an anterior portion of a rostrum, including premaxillae –with a premaxillary crest– and maxillae, both with alveoli and broken teeth.

#### Horizon and locality

Los Quiñones site, Morenillo limestones of the Blesa Formation, Barremian (Lower Cretaceous), Oliete sub-basin, Iberian Basin^12–14^. Obón, Teruel Province, Aragón, northeast Spain.

#### Diagnosis

Hamipterid pterodactyloid with the following autapomorphies: relatively deep premaxillary tip, premaxillary crest with its anterior margin curvature at an angle of about 80°.

#### Comparative description

**Figure 2:**
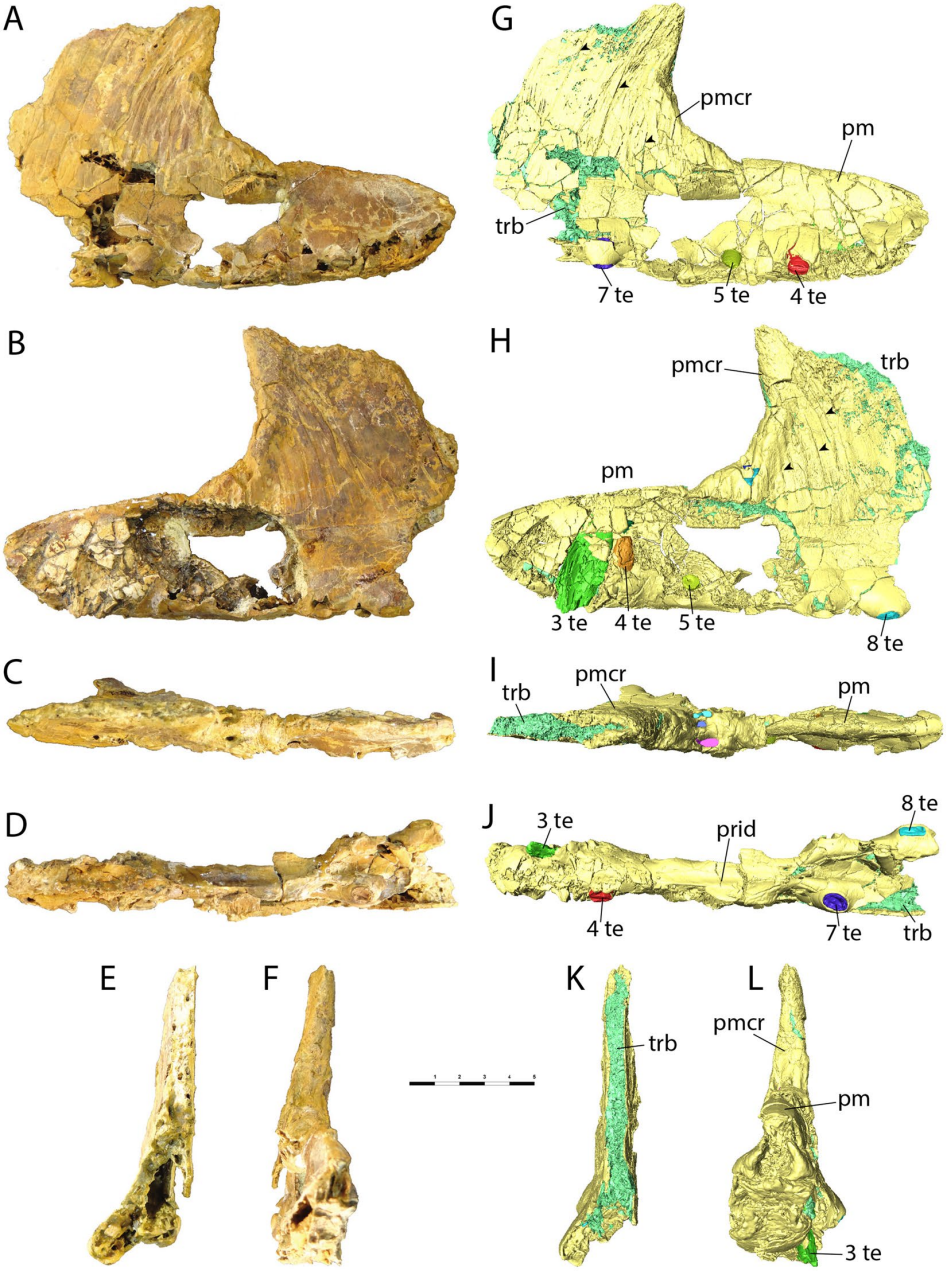
Partial rostrum of *Iberodactylus andreui* gen. et sp. nov. (MPZ-2014/1). Photographic images (**A**–**F**) and 3D renderings obtained from μCT data (**G**–**L**) in right lateral (**A**,**G**), left lateral (**B**,**H**), dorsal (**C**,**I**), palatal (**D**,**J**), caudal (**E**,**K**), and cranial (**F**,**L**) views. Scale bar in cm. Abbreviations. pm: premaxilla; pmcr: premaxillary crest; prid: palatal ridge; te: teeth; trb: trabeculae.

**Figure 3:**
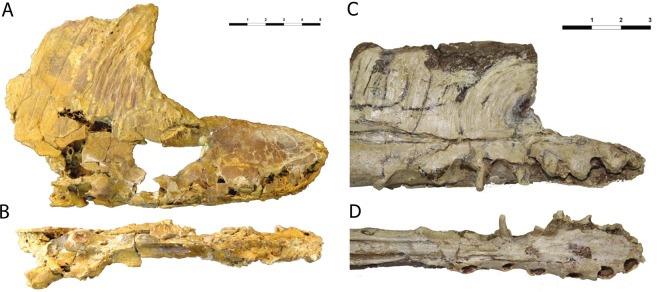
Comparison of the rostrum of *Iberodactylus andreui* gen. et sp. nov. (MPZ-2014/1) with a cast of a skull of *Hamipterus tianshanensis* (specimen stored at the Museu Nacional/Universidade Federal do Rio de Janeiro, Rio de Janeiro, Brazil (MN), MN-7536-V). Pictures in right lateral (**A**,**C**) and palatal (**B**,**D**) views.

The holotype (and so far only known material) of *Iberodactylus andreui* is a specimen (MPZ-2014/1) represented by a three-dimensional partial rostrum, including partial premaxillae and maxillae with a total preserved length of 199 mm and a height of 128 mm (Fig. 2). The premaxillary tip is expanded. The palate exhibits a palatal ridge and the anterior region is dorsally deflected. The alveoli are lateralized. A blade-like premaxillary crest starts approximately at the level of the fifth alveoli. The crest surface is covered by anteriorly curved striae and sulci, and its margin is anteriorly expanded. This combination is quite similar to what is seen in *Hamipterus tianshanensis* from the Berriasian-Albian of Xinjiang (NW China)^10,11^. However, MPZ-2014/1 differs from *Hamipterus* in having a quite deeper premaxillary tip. This comparison holds true even when ontogenetic variation is taken into consideration, as the premaxillary tips of both juvenile and adult specimens of *Hamipterus tianshanensis* present a relatively lower rostrum^11^. Also, the premaxillary crest differs from *Hamipterus* by the angle of curvature of the anterior margin (Fig. 3). Due to the robustness and height of the premaxillary crest, MPZ-2014/1 probably represents a male specimen as seen in the sexually dimorphic *Hamipterus*. This character is also different from all known ontogenetic series of *Hamipterus tianshanensis*^11^. Furthermore, the premaxillary crest starts at the fifth alveoli in both genera. It is however unclear if the same would apply to the new taxon due to the lack of other specimens so far. A micro-computed tomography (μCT) scan analysis revealed some tooth replacement and the position of the broken teeth within the premaxilla (see Supplementary Information for further details). An extreme trabecular web could be recognised inside the premaxillary crest. Three tiny asymmetrical holes with irregular shapes are recognised at the base of the premaxillary crest, which does not seem to be a natural anatomical feature.

Anhangueridae Campos & Kellner, 1985.

#### Node-based definition

The least inclusive clade containing *Anhanguera blittersdorffi*, *Coloborhynchus clavirostris* and *Tropeognathus mesembrinus*^18^.

#### Revised diagnosis

**Figure 4:**
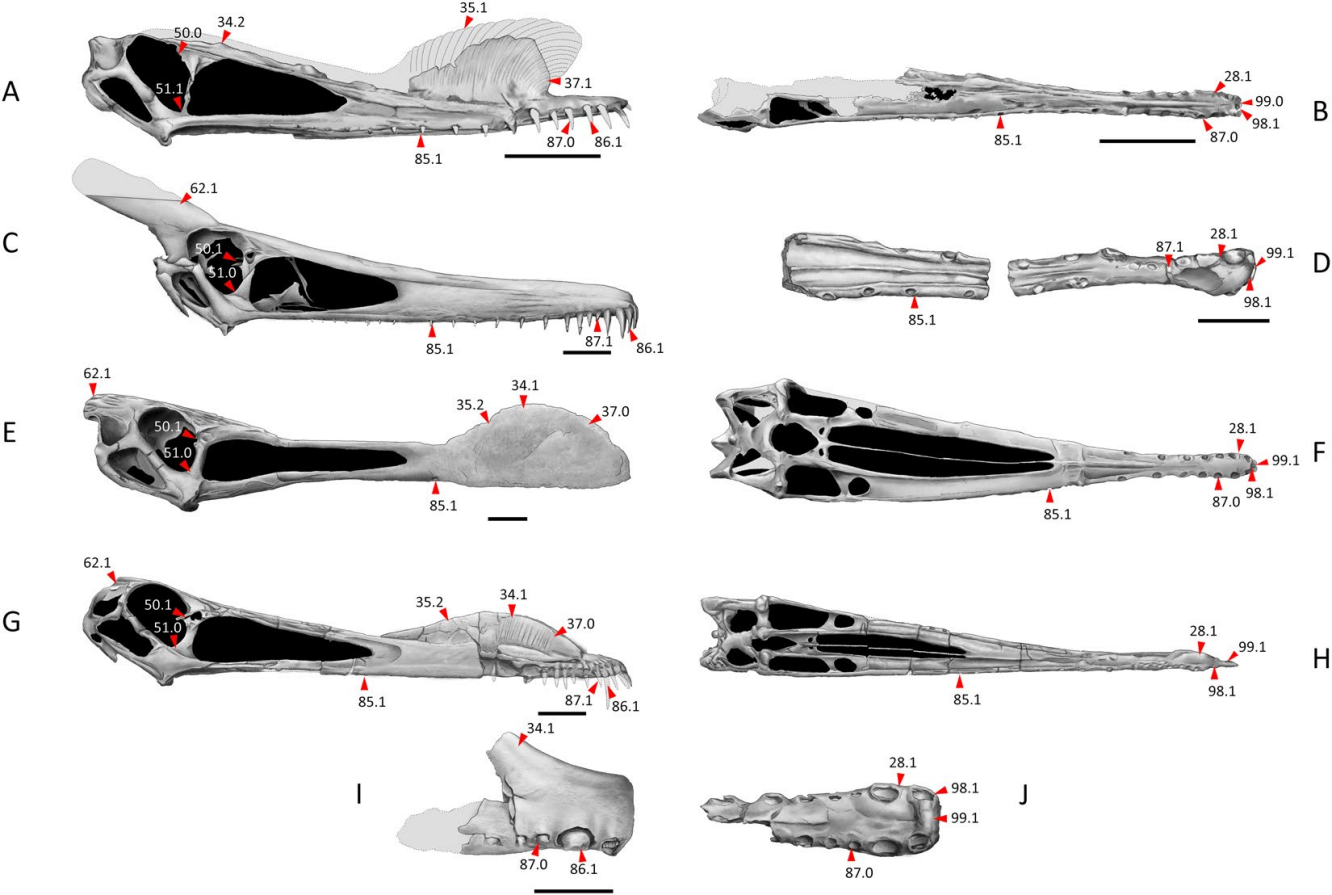
Skull characters of species from different lineages within Anhangueria. Each skull is based on the holotypes and paratypes (dark grey), and elements from other specimens (light grey) re-marked with broken lines. *Hamipterus tianshanensis* (IVPP V 18935.1), in righ lateral view (**A**) and palatal view (**B**) *Ludodactylus sibbicki* (specimen stored at the Staatliches Museum für Naturkunde Karlsruhe, Karlsruhe, Germany (SMNK), SMNK PAL 3828), in right lateral view (**C**) *Caulkicephalus trimicrodon* (specimen stored at the Isle of Wight County Museum Service, Sandown, Isle of Wight, England, United Kingdom (IWCMS), IWCMS 2002.189), in palatal view (**D**) *Tropeognathus mesembrinus* (specimen stored at the Bayerische Staatssammlung für Paläontologie und Geologie, Munich, Germany (BSP), BSP 1987 I 46), in right lateral view (**E**), and palatal view (**F**); *Anhanguera blittersdorffi* (MN 4805-V), in right lateral view (**G**), and palatal view (**H**) and *Uktenadactylus wadleighi* (specimen stored at the Southern Methodist University, Dallas, Texas, United States (SMU), SMU 73058), in right lateral view (**I**), and palatal view (**J**). Arrows show the character states in each skull. Scale bar 5 cm. See the Supplementary Information for details about number and state of characters.

Anhanguerians with markedly enlarged anterior teeth and the following exclusive combination of features: main part of the dorsal margin of the skull concave, premaxillary crest confined to the anterior portion of the skull; blade-shaped, rounded and smooth premaxillary crest; presence of an orbital process of the lacrimal; broad base of the lacrimal process of the jugal; dentary crest; and the first pair of upper alveoli located almost or entirely above the level of the second pair (instead of slightly raised as in *Cimoliopterus* and hamipterids). For further details see Fig. 4.

#### Content

Anhanguerinae, Coloborhynchinae and *Tropeognathus mesembrinus*.

#### Remarks

A dentary crest is unknown in coloborhynchines and *Cearadactylus*. Both premaxillary and dentary crests are absent in the holotype and only known specimen of *Guidraco venator* and unclear in *Ludodactylus sibbicki*^19,22,23^.

Anhanguerinae clade nov.

#### Stem-based definition

The most inclusive clade containing *Anhanguera blittersdorffi* but not *Coloborhynchus clavirostris*.

#### Diagnosis

Anhanguerids with an enlarged fourth premaxillary tooth, larger than the fifth and sixth teeth and as large as or larger than the third tooth.

#### Content

*Anhanguera, Caulkicephalus, Cearadactylus, Guidraco, Liaoningopterus, Ludodactylus and Maaradactylus*.

Coloborhynchinae clade nov.

#### Stem-based definition

The most inclusive clade containing *Coloborhynchus clavirostris* but not *Anhanguera blittersdorffi* or *Ludodactylus sibbicki*.

#### Diagnosis

Anhanguerids with a quadrangular expansion of the premaxillary tip and a flat anterior surface of the rostrum^24^.

#### Content

*Coloborhynchus, Siroccopteryx* and *Uktenadactylus*.

#### Phylogenetic analysis and comparison

In order to assess the phylogenetic position of *Iberodactylus andreui*, we performed a phylogenetic analysis using the software Tree analysis using New Technology (TNT) 1.5^25^. The analysis includes a broad sample of 17 anhanguerians –within a total of 55 pterosaur taxa– and 144 morphological characters. This analysis is based essentially on Vullo *et al*.^9^ (for further details see Supplementary Information) and resulted on 6 most-parsimonious trees (MPTs), with 336 steps, a consistency index of 0.67 and retention index of 0.87.

**Figure 5:**
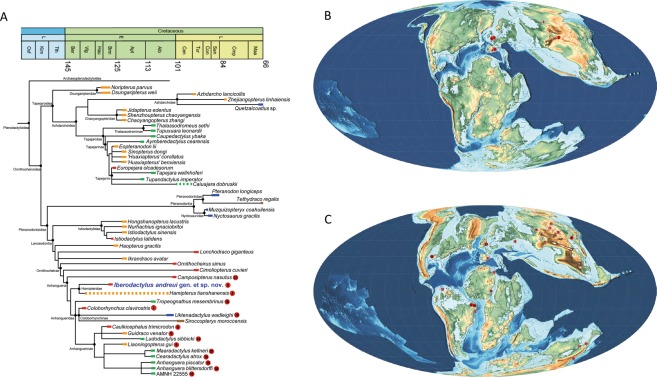
Origin and radiation of the clade Anhangueria during the Early Cretaceous. (**A**) Phylogenetic relationships of *Iberodactylus andreui* gen. et sp. nov. within Pterodactyloidea. Colours show their continental origin: Africa (brown), Asia (orange), Europe (red), North America (blue), and South America (green). Intermittent bars show uncertain temporal range; (**B**) Barremian world map showing the distribution of the localities with Anhangueria remains: (1) Hastings Group (late Berriasian/Valanginian), England; (2) Hami, Tugulu Group (?Berriasian-Albian), Xinjiang, China; (3) Bol’shoi Kemchug, lower Ilek Formation (?Hauterivian-Barremian) Krasnoyarsk Krai, Russia; (4) Las Hoyas, La Huérgina Formation (Barremian), Cuenca, Spain; (5) Los Quiñones, Blesa Formation (Barremian), Teruel, Spain; (6) Isle of Wight, Wessex Formation (Barremian), England; (**C**) Albian world map showing the distribution of the localities with Anhangueria remains: (7) Mogoito, Murtoi Formation (Aptian), Buryatia, Russia; (8) Sekmenevka Formation (Aptian), Belgorod Oblast, Russia; (9) Jiufotang Formation (Aptian), Liaoning, China; (10) Elrhaz Formation (Aptian), Niger; (11) Krasnyi Yar, Khilok Formation (Aptian), Buryatia, Russia; (12) Pedra Furada, Recôncavo Basin, Marizal Formation? (Aptian), Bahia, Brazil; (13) Sierra de Perijá, Apón Formation (Aptian), Zulia, Venezuela; (13) Crato Formation (late Aptian), Ceará, Brazil; (15) Khuren–Dukh, Dzun–Bayin Formation (Aptian-Albian), Mongolia; (16) Sheskatovo, upper Ilek Formation (Aptian-Albian), Kemerovo Oblast, Russia; (17) Chenini Formation (early Albian), Tunisia; (18) Romualdo Formation (Albian), Ceará, Brazil; (19) Lightning Ridge, Griman Creek Formation (Albian), New South Wales, Australia; (20) Tarrant County, Paw Paw Formation (Albian), Texas, USA; (21) Boulia, Toolebuc Formation (Albian), Queensland, Australia; (22) Cortes de Arenoso, Utrillas Formation (Albian), Valencia, Spain; (23) Cambridge Greensand (Cenomanian, but fossils Albian in age), England; (24) Hughenden, Mackunda Formation (late Albian), Queensland, Australia. Rose indicates purported remains associated within the clade Anhangueria. Red indicates taxa (referenced each one in A) within the clade Anhangueria. Palaeogeographic world maps modified after PALEOMAP Project (www.scotese.com).

*Hamipterus tianshanensis* and *Iberodactylus andreui* gen. et sp. nov. were found to form a monophyletic group in all trees (Fig. 5), the Hamipteridae fam. nov., sharing strong, well-defined concentric striae on the anterior region of the premaxillary crest, and an anterior expansion of the anterior margin of the premaxillary crest. This new family falls within the Anhangueria, sharing with other anhanguerians the presence of a lateral expansion on the rostral tips.

## Discussion

We have recovered *Iberodactylus andreui* gen. et sp. nov. in a sister-group relationship with *Hamipterus tianshanensis*, forming in turn a sister-group to the Anhangueridae. We further present here a new proposal for the phylogenetic relationships of the forms related to *Ornithocheirus* and *Anhanguera*. In our analysis, the least inclusive clade containing these genera –Ornithocheirae *sensu* Andres *et al*.^21^ –is supported herein by two synapomorphies, character state 94(1): the presence of long, slender teeth; and 95(1): the longitudinally striated tooth surface. We have recovered a clade composed of *Cimoliopterus* and the Anhangueria, supported by character state 98(1): a dorsally deflected palate. The Anhangueria *sensu* Rodrigues and Kellner^17^ is supported by character state 28(1): expansion of the premaxillary tip with the jaw end high; with *Camposipterus nasutus* at the base of the lineage. The clade formed by Hamipteridae + Anhangueridae is supported by character state 86(1): marked variation in size of the anterior teeth; the anterior teeth are quite large in these forms, unlike *Camposipterus nasutus* (Fig. 4). The Anhangueridae are set apart from the Hamipteridae on the basis of characters states 22(1): main part of the dorsal margin of the skull concave; 34(0): premaxillary crest confined to the anterior portion of the skull; 35(2): blade-shaped, rounded premaxillary crest; 50(1): presence of an orbital process of the lacrimal; 51(0): broad base of the lacrimal process of the jugal; 79(1) dentary crest; and 99(1) the first pair of upper alveoli located almost or entirely above the level of the second pair (instead of slightly raised as in *Cimoliopterus* and hamipterids). See the Supplementary Information and Fig. 4 for more details on the internal relationships of the Anhangueridae.

The Hamipteridae fam. nov. can be diagnosed by at least two synapomorphies: the presence of strong, well-defined parallel and forward curved striae and sulci on the anterior region of the premaxillary crest – character state 38(1) –, and the presence of a rounded anterodorsal expansion of the anterior margin of the premaxillary crest – character 39(1). Other crested lanceodontians lack these features, exhibiting smooth premaxillary crests with rounded margins as seen in anhanguerids or with a fine sculptured surface texture as seen in *Cimoliopterus*. Prior to the present recognition of the Hamipteridae and their classification as anhanguerians, Rodrigues and Kellner^17^ diagnosed the Anhangueria by the presence of both the lateral expansion of the rostrum and the enlarged anterior teeth. However, we now propose the markedly enlarged anterior teeth as diagnostic of a less inclusive group, excluding *Camposipterus nasutus*.

The sister-group of the Anhangueria is *Cimoliopterus*, sharing the presence of a premaxillary crest and character state 98(1): a dorsally deflected palate. The sister-group to Anhangueria + *Cimoliopterus* is, in our analysis, *Ornithocheirus simus*, which lacks a dorsally deflected palate (a palatal ridge was coded as unknown, following^17^).

Although the type and only known specimen of *Iberodactylus andreui* is very incomplete, more complete material of *Hamipterus tianshanensis* allows us to estimate its wingspan. Based on the proportions of *Anhanguera piscator*^26^, *Anhanguera* sp. (specimen stored at the American Museum of Natural History, New York City, United States (AMNH), AMNH 22555)^27^, and AMNH 22552^28^, we estimate that the large skull stored at the Institute of Vertebrate Paleontology and Paleoanthropology, Chinese Academy of Sciences, Beijing, China (IVPP), IVPP V 19831.3, must have had a wingspan of ~3.22 meters. Scaling this to the proportions of the holotype of *Iberodactylus andreui* results in a wingspan of ~4 meters.

The presence of an anhanguerian in the Barremian of the Iberian Peninsula is not surprising, since this group has been recorded elsewhere in the Early Cretaceous of Europe, such as the late Berriasian/Valanginian *Coloborhynchus clavirostris* from the Hastings Group, the Barremian *Caulkicephalus trimicrodon* from the Wessex Formation, and the Albian *Camposipterus nasutus* from the Cambridge Greensand^17^. However, *Iberodactylus andreui* gen. et. sp. nov is not closely related to any known European anhanguerian. The discovery of a sister-species to the Chinese form *Hamipterus tianshanensis* was indeed unexpected for the Iberian Peninsula. Their ages are coherent with this relationship, with *Hamipterus* coming from the Tugulu Basin dated as Berriasian-Albian^11,29–31^. The affinities of *Iberodactylus andreui* are well-supported by the presence of a palatal ridge (present in lanceodontians), a premaxillary crest, a dorsally deflected palate (present in *Cimoliopterus* and anhanguerians), a lateral rostral expansion (present in all anhanguerians), and markedly enlarged anterior teeth (shared by hamipterids and anhanguerids, though in anhanguerids such enlargement is even more pronounced than in hamipterids). The sister-group relationship between *Iberodactylus andreui* and *Hamipterus tianshanensis* is well-supported by two uncommon features of their premaxillary crests, unseen in any other lanceodontians. In this way, the discovery of *Iberodactylus andreui* confidently represents the presence of a sister-taxon to *Hamipterus tianshanensis* in Europe during the Early Cretaceous, adding to the list of related taxa between Europe and China during this time. This list also includes tapejarines (represented by *Europejara* and *Bakonydraco*^21^ in Europe and *Sinopterus*, ‘*Huaxiapterus*’ and *Eopteranodon* in China, even though *Europejara* is closer to Brazilian forms than to Asian taxa^9^) as well as the anhanguerines *Caulkicephalus trimicrodon* from England (Barremian) and *Guidraco venator* from the Aptian of Jiufotang Formation (China).

Apart from pterosaurs, other tetrapod lineages are recorded in the Iberian Peninsula with close affinities to Asian faunas^32–34^. The record of the terrestrial vertebrate faunas had shown Asian-related taxa in the Iberian Peninsula, even in close peer localities where *Iberodactylus andreui* gen. et. sp. nov was recovered^34^. Three remarkable sites with Asian-related forms are known for the Early Cretaceous of the Iberian Peninsula: the Berriasian site of Tera (Tera Group)^35^, and the Barremian sites of La Cantalera (Blesa Formation)^34^, Las Hoyas (La Huérgina Formation)^33^, and Vallipón (Artoles Formation)^36^. Fragmentary material of Titanosauriformes with close affinities to Asian taxa were found in Tera^35^. La Cantalera site includes titanosauriform and crocodyliform records related to Asian forms^32,34^. In the Vallipón site a gobiconodontid mammal was found^36^. The Las Hoyas *Lagerstätte* includes a diverse Asian-related fauna comprising crocodyliforms (related to *Gobiosuchus*)^33^; *Pelecanimimus* related to Asian ornithomimosaurs such as *Harpymimus* and *Garudimimus*^37^; enanthiornitean birds, including *Concornis lacustris* and *Eoalulavis hoyasi* closely related to *Qiliania*, *Gobipteryx* and *Vescornis*^38^; and the gobiconodontid mammal *Spinolestes xenarthrosus* related to *Gobiconodon* and *Repenomamus*^39^.

The discovery of *Iberodactylus* strongly suggests that the clade Anhangueria could have important ancestral ties in eastern Laurasia: the oldest records of this lineage come from the European archipelago and eastern Asian province (Fig. 5B), while the Aptian-Albian record was extended worldwide (Fig. 5C). Even though there were already known purported indeterminate anhanguerian teeth in the Iberian Peninsula^40^, *Iberodactylus* strengthens the diversity of anhanguerians at the beginning of the Early Cretaceous, constituting a better identifiable form from the Barremian European archipelago. Being *Coloborhynchus clavirostris* the oldest anhanguerian specimen known (Hastings Group, late Berriasian/Valanginian) and *Hamipterus tianshanensis* playing an uncertain role due to the vagueness of the Tugulu Group datation, *Iberodactylus andreui* emphasises an eastern Laurasian origin of the clade Anhangueria, as its contemporary anhanguerids from the Wessex Formation^17,41^. This clade spread during the Aptian, where its presence was not limited to Laurasia (with taxa such as *Guidraco venator* and *Liaoningopterus gui* from the Jiufotang Formation^19,42^) but also to northern Gondwana (with *Ludodactylus sibbicki* in Crato Formation^43^, but also some purported anhanguerian remains in other Brazilian localities^44^, Niger^45^ and Venezuela^46^). In the Albian record the anhanguerid diversity of the Romualdo Formation stands out^22,23^, as well as the presence of a taxon in North America (*Uktenadactylus wadleighi* from the Paw Paw Formation^24^), the Cambridge Greensand fauna^17^, and purported remains from Australia^47^. Thus, at the end of the Early Cretaceous the anhanguerians reached a worldwide distribution.

## Methods

### μCT scan analysis

The specimen MPZ-2014/1 was scanned at the Centro Nacional de Investigación sobre Evolución Humana (CENIEH, Burgos, Spain) using a high-resolution x-ray tomography scanner μCT model V|Tome|X s 240 (GE Sensing & Inspections Technologies). The specimen was scanned at 160 kV and 200 μA using a filter of 0.2 mm of Cu and obtaining 7.5 μm of voxel size. The μCT data of the scanned specimen was imported to the software Avizo 7.0 (FEI-VSG company), where a reconstruction and segmentation were performed, and bone, teeth and matrix were separated in layers to analyse inner bone structures.

### Nomenclatural acts

This published work and the nomenclatural acts it contains have been registered in ZooBank, the proposed online registration system for the International Code of Zoological Nomenclature. The ZooBank Life Science Identifiers (LSIDs) can be resolved and the associated information viewed by appending the LSIDs to the prefix http://zoobank.org/. The LSID for this publication is urn:lsid:zoobank.org:pub:AD09BA44-4BC3-49ED-B5AE-53D9B343CC6E, and the LSIDs for the new erected groups and taxa are: urn:lsid:zoobank.org:act:CBBB6FBB-54A0-4E0B-99C6-2D3E6BC2A110 (Hamipteridae), urn:lsid:zoobank.org:act:15EB1E14-3C13-4F5A-A290-6A046F7967ED (Anhanguerinae), urn:lsid:zoobank.org:act:146AE3A1-AC77-4495-9558-4FD4E31BFE40 (Coloborhynchinae), urn:lsid:zoobank.org:act:0174E98C-416B-4C49-AF63-2B42AF1E9EAB (*Iberodactylus*), and urn:lsid:zoobank.org:act:37FAC334-082A-4185-970E-7E7E13D5670C (*Iberodactylus andreui*).

### Phylogenetic analysis

We performed a phylogenetic analysis using the software TNT 1.5^25^. This analysis is based essentially on Vullo *et al*.^9^ (for further details see dataset file in the Supplementary Information). Search for the most parsimonious trees (MPTs) was conducted via Traditional Search (TBR swapping algorithm), 10,000 replicates, random seed and collapsing trees after search.

### Wingspan estimation

In order to produce an estimate for the wingspan of the holotype of *Iberodactylus andreui*, we propose here, firstly, estimates for *Hamipterus tianshanensis*. In the single block IVPP V 18931, a large skull of *H*. *tianshanensis* and a partial wing were found in association^9^. The distance between the first and sixth pairs of upper alveoli is 113.8 mm in this skull (see Supplementary information). The same distance is 11.9 cm in *Iberodactylus andreui*. We have estimated the total wingspan of the partial *Hamipterus* wing based on the more complete material of *Anhanguera piscator*^26^, *Anhanguera* sp. (AMNH 22555)^27^, and AMNH 22552^28^. We thus scaled these wingspan estimations from the distance between the first and sixth pairs of upper alveoli in *H*. *tianshanensis* to the same distance in *Iberodactylus andreui* in order to obtain an estimate for the latter. See Supplementary Material for a table with all the measurements of *Anhanguera piscator*, *Anhanguera* sp., AMNH 22552 and IVPP V 18931.3.

## Supplementary information

Supplementary Information

Dataset 1
