# Supplementary material for: On a new crested pterodactyloid from the Early Cretaceous of the Iberian Peninsula and the radiation of the clade Anhangueria
Source: Sci Rep. 2019 Mar 20;9:4940. doi: 10.1038/s41598-019-41280-4 (PMC6426928; doi:10.1038/s41598-019-41280-4)
Supplement: Supplementary file 1 — Supplementary Information [file 41598_2019_41280_MOESM1_ESM.pdf]

On a new crested pterodactyloid from the Early Cretaceous of the Iberian Peninsula and the radiation of the clade Anhangueria

Borja Holgado[1,2*], Rodrigo V. Pêgas[1], José Ignacio Canudo[3,4], Josep Fortuny[2], Taissa Rodrigues[5], Julio Company[6], Alexander W. A. Kellner[1]

[1]Laboratory of Systematics and Taphonomy of Fossil Vertebrates, Departmento de Geologia e Paleontologia, Museu Nacional/UFRJ, Rio de Janeiro, Brazil. [2]Institut Català de Paleontologia 'Miquel Crusafont', Universitat Autònoma de Barcelona, Cerdanyola del Vallès, Catalonia, Spain. [3]Grupo Aragosaurus-IUCA, Área de Paleontología, Facultad de Ciencias, Universidad de Zaragoza, Zaragoza, Spain. [4]Museo de Ciencias Naturales de la Universidad de Zaragoza, Zaragoza, Spain. [5]Laboratório de Paleontologia, Universidade Federal do Espírito Santo, Vitória, Brazil. [6]Departamento de Ingeniería del Terreno, Universidad Politécnica de Valencia, Valencia, Spain. Correspondence and request for materials should be addressed to B.H. (email: borja.holgado@mn.ufrj.br)

Extended description

The specimen MPZ-2014/1 consists of a partial rostrum of approximately 198 mm in length, composed of the anterior part of the premaxilla with a partially preserved premaxillary crest, and a fragment of the maxillary bone including several fragmentary teeth (Fig. 2). The specimen preserves its original three-dimensional shape, although it is somewhat crushed, partly eroded, and several broken bone fragments are lost. The posterior transverse section of the specimen is somewhat triangular in outline, whereas the anterior region exhibits a compressed contour. The frequent fractures of the specimen and the eroded bone surfaces reveal an external thin layer of cortical bone of 1.5 mm, enclosing a delicate trabecular tissue.

Premaxilla. The premaxilla is represented by an anterior fragment of the bone comprising the premaxillary tip up to the posterior broken border, a little behind the

level of the eight alveoli. The anterior dorsal margin of the premaxilla is not straight, but slightly curved.

There is no clear suture between the premaxillae and the maxillae, even though a faint groove could be recognized, which has been interpreted as a suture in other anhanguerian pterodactyloids[26]. This would allow interpreting that the premaxillae and the maxillae are fused, in this case up to the position of the 5th alveoli[26].

The most striking feature is the presence of a pronounced premaxillary crest whit a strongly concave anterior border. The crest starts at the level of the fifth alveoli. The backward extension of the crest is unknown due to the fragmentary nature of the specimen. The crest is triangular in cross-section. The crest exhibits well-developed elongate sub-vertical striae and sulci, anteriorly curved (Fig. 2G-H), similar to those observed in the maxillary crest of *Hamipterus tianshanensis*[10]. Such striae are present in other pterosaurs[4,48-50], but the combination with sulci is exclusively preserved –apart from the Hamipteridae– in the Triassic pterosaur *Raeticodactylus filisurensis*[4,50]. In this pterosaur it was suggested that the suture-like structure, visible between the premaxillary crest and the premaxillae-maxillae, may have separated the soft tissue part from the rest of the skull[49]. This structure seems to be similar in shape and anatomical position to the faint groove observed in *Iberodactylus andreui*, which presents the same depth of the premaxillary crest sulci. Such sulci could be interpreted as a trait related to the attachment of the rhamphotheca, as in the case of some extant birds[51].

Regarding the tiny holes located at the anterior margin of the premaxillary crest, their nature is unclear, but some considerations can be made. Firstly, there is no evidence to indicate these structures are originally part of the specimen. They are asymmetrical in number, shape, and position. Furthermore, they do not seem to be of pathological nature as the μCT scan revealed no bone remodelling, resorption, or

changes in density (Fig. S1). Therefore, their origin is more likely to be taphonomic in nature. Though hard to ascertain, we suspect that action of osteophagic organisms could be a likely explanation[52-55]. Nevertheless, the preservation of cortical bone at the surface of the holes –even though this tissue is not present internally– call this interpretation into question.

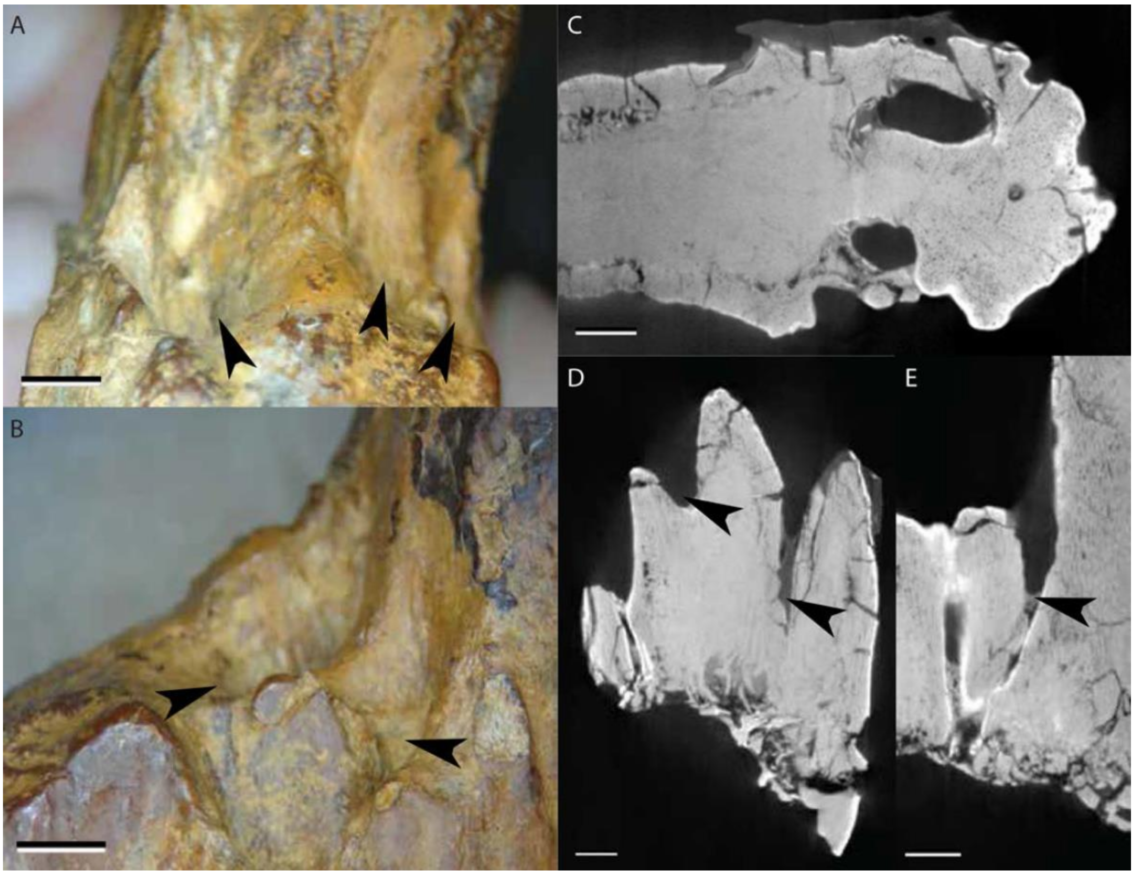

Figure S1. Detailed pictures of the holes located at the base of the premaxillary crest of *Iberodactylus andreui* gen. et sp. nov. (A) cranio-dorsal; and (B) latero-dorsal view. μCT scan slices with a detail of the holes in (C) dorso-ventral; (D) cranio-caudal; and lateral (E) views. Arrows indicate the accurate location of the holes. Scale bar = 2 mm.

Maxilla. The maxilla is only partially known by a fragment comprising its anteriormost part, extending backwards behind the level of the 7th and 8th alveoli. Albeit with some distortion, the bone preserves most of its three-dimensional shape. It is noticeably

crushed at the level of the anterior alveoli, and some of them have been badly eroded along the lateral sides of the bone. Part of the palatal plate is preserved in the specimen.

Alveolar row and teeth. Up to fifteen complete or fragmentary dental alveoli are visible on the ventrolateral edge of the rostrum. Alveolus one to eight are preserved on the right side of the specimen, whereas the alveoli one to five, and the seventh and eighth ones, can be recognized on the left side. The sixth alveolus of the left side has been completely eroded away. The first two alveoli are placed on the anterior margin of the rostrum and seem to be forwardly directed. The remainder alveoli face laterally. Spacing between alveoli is slightly concave and the alveolar lateral walls are clearly convex. They are small and elliptical in shape, between 5 and 9 mm in diameter labio-lingually and between 9 and 20 mm anteroposteriorly (See Table S1).

Teeth are set in individual sockets, and not all of them are completely exposed. Dentition seems heterodont in size and shape, as there is a noticeable variation of basal crown measurements along the tooth row (Table S1). The bases of the crowns are elliptical in shape with the long axis parallel to the sagittal plane of the bone. All the teeth preserved in situ have their crowns broken. They correspond to the right fourth, fifth and seventh teeth, with the eighth tooth on the left side.

Table S1. Alveolar scheme for *Iberodactylus* rostrum. Abbreviations: R, right alveolus; L, left alveolus; T-, broken tooth; Rt, root only. Length, mesiodistal diameter of the alveoli; width, labiolingual diameter of the alveoli (measurements in mm).

**Alveoli**	**1R**	**1L**	**2R**	**3R**	**4R**	**5R**	**6R**	**7R**	**7L**	**8L**
**Length**	9.6	9.8	15.0	20.0	9.6	11.0	16.0	8.7	9.0	8.6
**Width**	5.1	5.0	8.2	9.5	6.5	6.3	7.20	6.0	7.5	5.6
**Tooth**	-	-	-	-	T-	T-	-	Rt	-	Rt

Phylogenetic analysis

In order to assess the phylogenetic position of *Iberodactylus andreui*, we performed a phylogenetic analysis using the software TNT 1.5[25] using the TBR heuristic searches performed using maximum parsimony. Characters were given equal weight and treated unordered. This analysis is based essentially on Vullo et al.[9]. Search for the most parsimonious trees (MPTs) were conducted via Traditional Search (TBR swapping algorithm), 10,000 replicates, random seed, 20 trees to save per replication, and collapsing trees after search. The search conducted by TNT including all three outgroups (*Ornithosuchus woodwardi*, *Herrerasaurus ischigualastensis* and *Scleromochlus taylori*) resulted 6 most-parsimonious trees (MPTs) and a strict consensus tree with a length of 336 steps (consistency index = 0.67; retention index = 0.87) (Fig. S1).

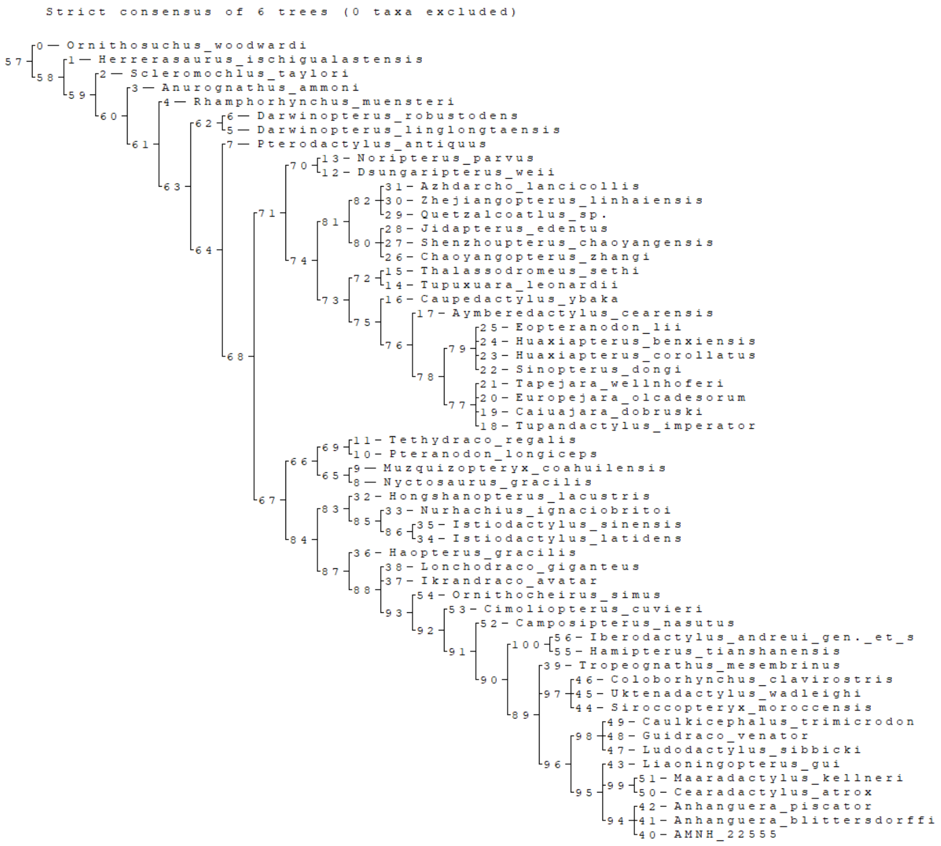

Figure S2. Strict consensus tree of the phylogenetic relationships of *Iberodactylus andreui* gen. et sp. nov. within Pterodactyloidea (Pterosauria, Ornithodira).

Character list (per anatomical region)

CRANIUM (SKULL)

001. External naris (or nasoantorbital fenestra), position relative to the premaxilla (Andres et al.[21]: character 48; modified from Kellner[56]: character 5):

0 - main part dorsal to the ventral margin of the premaxilla

1 - main part displaced posterior to the premaxilla

002. External naris, dorsoventrally compressed:

0 - absent

1 - present

003. External naris and antorbital fenestra, configuration (rephrased from Unwin[57]):

0 - separated

1 - confluent, forming a nasoantorbital fenestra

004. External naris and antorbital fenestra (or nasoantorbital fenestra), ventral margin length relative the skull length (rephrased from Kellner[56]: character 7):

0 - shorter than 40% of the skull length

1 - longer than 40% of the skull length

005. Antorbital (or nasoantorbital) fenestra, posterior margin, shape (modified from Unwin[57]):

0 - straight

1 - concave

006. Nasoantorbital (or antorbital) fenestra extending dorsal to the orbit:

0 - absent

1 - present

007. Orbit, shape (modified from Kellner[58]: character 10; combined with Andres & Ji[59]: character 9):

0 - subcircular

1 - quadrangular (broad base)

2 - piriform (dorsoventrally elongated)

008. Orbit, comparatively small:

0 - absent

1 - present

009. Ventral margin of the orbit:

0 - closed

1 - open

010. Orbit, position (Andres & Myers[60]: character 45; modified from Kellner & Langston[61]; Kellner[56]: character 9; and Andres & Ji[59]: character 10):

0 - middle of the skull, with the ventral margin of the orbit below the middle of the antorbital (or nasoantorbital) fenestra and the dorsal margin of the orbit above the dorsal margin of the antorbital (or nasoantorbital) fenestra

1 - high in the skull, with the ventral margin of the orbit the same level or above the middle of the antorbital (or nasoantorbital) fenestra

2 - low in the skull, with the entire orbit lower than the dorsal margin of the antorbital (or nasoantorbital) fenestra

011. Suborbital opening (Kellner[56]: character 10):

0 - absent

1 - present

012. Lower temporal fenestra, shape (Wang et al.[19]: character 15):

0 - comparatively broad, with extensive subhorizontal ventral margin (trapezoidal)

1 - piriform, with dorsal portion wider than ventral

2- piriform, with ventral portion wider than dorsal

3 - reduced (slit-like)

013. Lower temporal fenestra, position relative to orbit (Andres et al.[21]: character 57):

0 - posterior to orbit

1 - reaches under posterior margin of orbit

014. Choanae, separation:

0 - separated by vomer

1 - confluent

015. Postpalatine fenestra, shape:

0 - quadrangular/subriangular

1 - oval

2 - egg-shaped

3 - elongated egg-shaped

4 - kite-shaped, rounded margins

5 - elliptical

6 - reduced, slit-like

016. Secondary subtemporal fenestra:

0 - absent

1 - present

017. Interpterygoid fenestra, size (modified from Kellner[56]: character 26):

0 - smaller than subtemporal fenestra

1 - larger than subtemporal fenestra

2 - extremely reduced

018. Interpterygoid fenestra, shape:

0 - compressed laterally

1 - broad, longer than wide

2 - compressed anteroposteriorly, wider than long

3 - round

019. Pterygoid fenestra:

0 - absent

1 - present

020. Upper and lower jaw, marked gap during occlusion:

0 - absent

1 - present

021. Upper and lower jaw, shape:

0 – laterally compressed

1 – comparatively broad

022. Skull, main part of dorsal margin, curvature excluding cranial crest (modified from Kellner[56]: character 3):

0 - nearly straight

1 - concave
2 - convex

023. Length of the rostrum (pm-naof) relative to the skull length (pm-sq) (modified from Kellner[56]: character 1):

0 - reduced

1 - elongated (about or less than half of skull length)

2 - extremely elongated (more than half of skull length)

024. Rostrum, anterior end (premaxilla), shape:

0 - rounded

1 - pointed

2 - sharp tip

3 - flat surface

025. Rostral end of premaxillae/maxillae downturned:
0 - absent

1 - present

026. Rostrum, distinct concavity on occlusal surface:

0 - absent

1 - present

027. Rostrum, anterior portion forming a high ossified plate:

0 - absent

1 - present

028. Premaxillae, anterior horizontal expansion (modified from Kellner[56]: character 12):

0 - absent
1 - present, with premaxillary end high
2 - present, with premaxillary end dorsoventrally flattened

029. Premaxillae anterior expansion, shape in horizontal plane (Andres & Myers[60]: character 37; modified from Kellner[56]: character 12):

0 - elliptical

1 - anteriorly expanded

2 - quadrangular

3 - absent

030. Premaxillae, anterior end rodlike:

0 - absent

1 - present

031. Premaxillary process separating the external nares, thickness (Kellner[58]: character 2):

0 - wide

1 - narrow

032. Premaxilla, posterodorsal margin of nasoantorbital fenestra (including nasal), width (modified from Andres et al.[21]: character 58; rephrased from Lü et al.[62]: character 8):

0 - wide

1 - thin

033. Premaxillary sagittal crest (modified from Kellner[18]: character 12):

0 - absent

1 - present

034. Premaxillary sagittal crest, position (modified from Kellner[18]: character 12):

0 - confined to the anterior portion of the skull

1 - starting anterior to the anterior margin of the nasoantorbital fenestra, extending beyond occipital region

2 - starting at about the anterior margin of the nasoantorbital fenestra, reaching the skull roof above the orbit but not extending over the occipital region

3 - starting close or at the anterior portion of the skull and extended over the occipital region

4 - starting at the posterior half of the nasoantorbital fenestra.

5 - starting at the middle portion of the nasoantorbital fenestra, extending above the occipital region

035. Premaxillary sagittal crest, shape (Andres & Myers[60]: character 52):

0 - striated, low with a nearly straight dorsal margin

1 - striated, high with a nearly straight dorsal margin

2 - round dorsal margin, blade-shaped

3 - smooth, expanded anteriorly and forming a low rod-like extension posteriorly

4 - smooth, starting low anteriorly and very expanded posteriorly

5 - striated, low with convex dorsal margin

036. Premaxillary crest, elongated dorsal premaxillary spike-like projection (rephrased from Martill & Naish[63]: character 2):

0 - absent

1 - present

037. Premaxillary crest, distinct expansion on the anterior part (Pinheiro et al.[64]: character 9):

0 - absent

1 - present

038. Premaxillary crest, well-defined parallel and forward curved striae and sulci on the anterior region (new character):

0 - absent

1 - present

039. Premaxillary crest, anterior margin, strong re-entrancy at the base (new character):

0 - absent

1 - present

040. Premaxilla, posterior dorsal process, curved upward:

0 - absent

1 - present

041. Maxilla, posterior ventral expansion (Kellner[56]: character 13):

0 - absent

1 - present

042. Maxilla-nasal contact (modified after Unwin[65]: character 13):

0 - absent

1 - present

043. Maxilla-nasal contact, broadness (Unwin[65]: character 13):

0 - broad

1 - narrow

044. Nasal descending process, position (modified after Kellner[56]: character 14):

0 - absent

1 - present

045. Nasal descending process, position (modified after Kellner[56]: character 14):

0 - placed laterally

1 - placed medially

046. Nasal descending process, length (modified after Kellner[18]: character 15, Andres & Ji[59]: character 23):

0 - long, almost reaching the ventral margin of the skull

1 - short

2 - knob-like (extremely reduced)

047. Nasal descending process, orientation (modified after Kellner[18]: character 15):

0 - inclined anteriorly

1 - subvertical

048. Nasal descending process, lateral foramen (rephrased from Kellner[18]: character 16):

0 - absent

1 - present

049. Lacrimal, extensive fenestration:

0 - absent

1 - present

050. Lacrimal, orbital process (modified from Andres & Myers[60]: character 66):

0 - absent

1 - present

051. Jugal, lacrimal process base, width (modified from Kellner[58]: character 18):

0 - broad

1 - narrow

052. Jugal, lacrimal process, inclination (modified from Kellner[58]: character 18):

0 - inclined anteriorly

1 - subvertical

2 - inclined posteriorly

053. Jugal, presence of pronounced ridge on the lateral side:

0 - absent

1 - present

054. Jugal, postorbital process, orbital process (Andres et al.[21]: character 98):

0 - absent

1 - present

055. Quadrate, inclination relative to ventral margin of skull (based on Unwin[57]; rephrased from Kellner[56]: character 18):

0 - anteriorly

1 - subvertical

2 - inclined about 120° posteriorly

3 - inclined about 150° posteriorly

056. Cranio-mandibular articulation, position relative to orbit (modified from Kellner[56]: character 19):

0 - posterior to posterior margin of orbit

1 - under center of orbit

2 - under anterior margin of the orbit

3 - anterior to the anterior margin of orbit

057. Helical jaw joint (Bennett[66]: character 5):

0 - absent

1 - present

058. Frontal, anterior portion rugose (Wang et al.[67]):

0 - absent

1 - present

059. Frontal, ossified crest (Bennett[66]: character 30):

0 - absent

1 - present

060. Frontal, ossified crest, position (Wang et al.[19]):

0 - confined to posterior end of skull
1 - starting above orbit
2 - starting on posterior half of nasoantorbital fenestra

061. Frontal, ossified crest, shape (modified from Kellner[56]: character 15)

0 - low, blunt
1 - short, spike-like, dorsally deflected

2 - spike-like, directed posteriorly

3 - narrow, broad, directed posteriorly
4 - low, broad base, fan-shaped

5 - high, broad base, crown-shaped

6 - high, broad base, casqued-shaped

7 - high, broad base, directed posteriorly, at least doubling height of skull above orbit.

062. Parietal, ossified crest (modified from Kellner[56]: character 16):

0 - absent
1 - present

063. Parietal, ossified crest, shape (modified from Kellner[56]: character 16):

0 - blunt
1 - constituting the base of the posterior portion of the cranial crest

2 - expanded, with rounded margin

064. Posterior region of the skull rounded with the squamosal displaced ventrally:

0 - absent
1 - present

065. Supraoccipital (Kellner[56]: character 20):

0 - does not extend backwards

1 - extends backwards

066. Supraoccipital, foramen (rephrased from Kellner[56]: character 21):

0 - absent

1 - present

067. Paroccipital processes, expanded distal ends (rephrased from Unwin[57]):

0 - absent

1 - present

068. Foraminae piercing the anterior portion of the palate, numerous:

0 - absent

1 - present

069. Palatal occlusal surface (modified from Kellner[56]: character 24):

0 - smooth

1 - discrete palatal ridge, tapering anteriorly
2 - strong palatal ridge, tapering anteriorly
3 - strong palatal ridge, confined to the posterior portion of the palate

070. Palate, slight expansion close to the anterior margin of the nasoantorbital (or naris + antorbital) fenestra:

0 - absent

1 - present

071. Palate, dorsal deflection (modified from Andres & Ji[59]; Rodrigues & Kellner[17]):

0 - absent

1 - present, slight; with the first pair of upper alveoli not completely surpassing the second pair

2 - present, strong; with the first pair of upper alveoli above the second pair

072. Maxilla and internal naris, (rephrased from Kellner[56]: character 25):

0 - contact

1 - do not contact

073. Palatines, shape (based on Unwin[65]: character 15):

0 - broad

1 - thin bars

074. Basisphenoid body, length (modified from Kellner[56]: character 23):

0 - shorter than wide

1 - longer than wide

MANDIBLE (Lower Jaw)

075. Mandibular rostral end, opposing dentaries (rephrased from Unwin[56]; Kellner[56]: character 27)

0 - unfused

1 - fused

076. Mandibular rostral end, extension of the contact surface of opposing dentaries (Kellner[56]: character 27; Pêgas et al.[68]: character 52):

0 - short, limited to the tip

1 - short, extended posteriorly less than 30% of mandible length

2 - long, up to 55% the mandible length

3 - long, extended over 55% of mandibular length

077. Mandibular rostral end, shape:

0 - rounded

1 - pointed

2 - sharp tip

078. Dentary, dorsal margin, distinct posterior eminence close to the separation of mandibular rami:

0 - absent

1 - present

079. Dentary, tip projected anteriorly:

0 - absent

1 - present

080. Dentary ossified sagittal crest (modified from Kellner[18]: character 33):

0 - absent

1 - present

081. Dentary ossified sagittal crest, position (modified from Kellner[18]: character 33):

0 - confined to the anterior third of the lower jaw

1 - extending close to the middle portion of the jaw

082. Dentary ossified sagittal crest, shape (modified from Kellner[56]: character 30):

0 - shallow

1 - blade-like

2 - deep, broad in lateral view

3 - elongated ridge

083. Dentary, posteroventral fossa: dentary fossa

0 - absent

1 - present

DENTITION

084. Teeth, position and presence:

0 - present, evenly distributed along the jaws
1 - absent from the anterior portion of the jaws
2 - confined to the anterior part of the jaws
3 - jaws toothless

085. Maxillary teeth, largest positioned posteriorly (rephrased from Unwin[57]):

0 - absent

1 - present

086. Teeth, shape variation:

0 - isodont

1 - heterodont

2 - absent

087. Teeth, anterior, marked variation in size:

0 - absent

1 - present

088. Teeth, upper jaw, variation in the size of the anterior teeth with the 4th larger than the 5th and 6th (modified from Kellner[56]: character 33):

0 - absent

1 - present

089. Teeth, base broad and oval (rephrased from Unwin[57]):

0 - absent

1 - present

090. Teeth, serrated:

0 - present

1 - absent

091. Teeth, peg-like (cone-shaped) (Kellner[56]: character 35):

0 - absent

1 - present, 15 or less on each side of the jaws

2 - present, more than 15 on each side of the jaws

092. Teeth, small needle-shaped:

0 - absent

1 - present

093. Teeth, laterally compressed and triangular:

0 - absent

1 - present

094. Teeth, sharp carinae:

0 - absent

1 - present

095. Teeth, elongated (modified from Kellner[18]: character 40):

0 - absent

1 - present

096. Teeth, fluted surface (modified from Andres & Myers[60]: character 110):

0 - absent

1 - present

098. Alveoli, lateral platform:

0 - absent

1 - present

AXIAL SKELETON

099. Atlas and axis:

0 - unfused

1 – fused

100. Cervical vertebrae, postexapophyses:

0 - absent

1 - present

101. Mid-cervical vertebrae, centrum, lateral foramen:

0 - absent

1 - present

102. Mid-cervical vertebrae, length:

0 - short, sub-equal in length

1 - longer than wide, with length less than 3 times width

2 - elongated, with length more than 3 times width

3 - extremely elongated

103. Mid-cervical vertebrae, ribs:

0 - present

1 - absent

104. Mid-cervical vertebrae, neural spines, height (Andres & Ji[59]: char. 67):

0 - tall

1 - low

2 - extremely reduced

105. Mid-cervical vertebrae, neural spines, shape (modified from Kellner[56]: character 43; Andres & Ji[59]: char 66):

0 - blade-shaped

1 - spike-shaped

2 - ridge

106. Dorsal vertebrae, fused into a notarium:

0 - absent

1 - present

107. Caudal vertebrae, quantity:

0 - more than 15

1 - 15 or less

108. Caudal vertebrae, zygapophyses forming rod-like ossified processes:

0 - absent

1 - present

109. Proximal caudal vertebrae centrum, centrum shape (rephrased from Bennett[66]: character 31):

0 - single

1 - duplex

PECTORAL GIRDLE

110. Scapula, length relative to coracoid length:

0 - subequal or longer than coracoid

1 - scapula shorter than coracoid (1 > sca/cor > 0.80)

2 - substantially shorter than coracoid (sca/cor $\leq$ 0.80)

111. Scapula, proximal end (rephrased from Kellner[18]: character 50):

0 - elongated

1 - sub-oval

112. Scapula, shape (rephrased from Kellner[18]: character 51):

0 - elongated

1 - stout, with constructed shaft

113. Coracoid, proximal end, shape:

0 - flattened

1 - oval

114. Coracoid, sternal articulation (modified from Kellner[18]: character 52):

0 - no developed articulation surface

1 - articulation straight or slightly concave

2 - articulation strongly concave

115. Coracoid, sternal articulation, posterior expansion:

0 - absent

1 - present

116. Coracoid, ventral margin, deep flange

0 - absent
1 - present

117. Coracoid, broad tubercle on ventroposterior margin (Kellner[58]: character 57):

0 - absent

1 - present

118. Cristospine, shape (rephrased from Bennett[66]: character 34):

0 - absent

1 - shallow and elongated

2 - deep and short

FORELIMB

119. Humerus, proportional length relative to the metacarpal IV (hu/mcIV) (Kellner[56]: character 46):

0 - hu/mcIV > 2.50

1 - 1.50 < hu/mcIV < 2.50

2 - 0.40 < hu/mcIV < 1.50

3 - hu/mcIV < 0.40

120. Humerus, proportional length relative to the femur (hu/fe) (modified from Kellner[56]: character 47):

0 - hu/fe ≤0.80

1 - 1.4 > hu/fe > 0.80

2 - hu/fe > 1.40

121. Humerus plus ulna, proportional lengths relative to the femur plus tibia (hu+ul/fe+ti) (Kellner[56]: character 48):

0 - humerus plus ulna about 0.80% or less of femur plus tibia length (hu+ul/fe+ti < 0.80)

1 - humerus plus ulna larger than 0.80% of femur plus tibia length (hu+ul/fe+ti > 0.80)

122. Humerus, proximal end, foramen on dorsal surface near medial margin (rephrased from Unwin[65]: character 34):

0 - absent

1 - present

123. Humerus, proximal end, foramen on ventral surface close to proximal margin (modified from Kellner[56]: character 49):

0 - absent

1 - present

124. Humerus, deltopectoral crest, shape (modified from Kellner[18]: character 58):

0 - reduced, positioned close to the humerus shaft

1 - enlarged, proximally placed, with almost straight proximal margin

2 - enlarged, hatchet shaped, proximally placed

3 - enlarged, hatched shaped, positioned further down the humerus shaft

4 - enlarged, warped

5 - long, proximally placed, curving ventrally

6 - enlarged, square outline

125. Humerus, medial (= ulnar) crest (modified from Kellner[56]: character 51):

0 - reduced

1 - directed posteriorly

2 - present, massive, with a developed proximal ridge

126. Humerus, distal articulation, shape (Andres & Myers[60]: character 159):

0 - oval or D-shaped
1 - subtriangular

127. Humerus, between distal condyles, pneumatic foramen (Longrich et al.[69]: character 227):

0 - absent

1 - present

128. Ulna, proportional length relative to metacarpal IV (ul/mcIV) (Kellner[18]: character 61; modified from Kellner[56]: character 53):

0 - ulna 3.6 times longer than metacarpal IV (ul/mcIV > 3.6)

1 - length of ulna between 3.6 and two times the length of metacarpal IV (3.6 > ul/mcIV > 2)

2 - ulna between two times and the same length of metacarpal IV (2 > ul/mcIV > 1)

3 - ulna about the same length or smaller than metacarpal IV (ul/mcIV < 1)

129. Ulna and radius, diameter at midshaft (Kellner[18]: character 62; modified from Bennett[66]: character 33)

0 - subequal

1 - diameter of radius about half that of ulna

2 - diameter of radius less than half that of ulna

130. Distal syncarpals, shape (distal view) (modified from Kellner[56]: character 55):

0 - irregular

1 - from rectangular unit

2 - form triangular unit

131. Pteroid:

0 - absent
1 - shorter than half the length of the ulna
2 - longer that half the length of the ulna

132. Pteroid, proximal articulation, expanded in right angle with the shaft:

0 - absent

1 - present

133. Metacarpals I - III, relation with carpus (modified from Bennett[66]: character 15):

0 - articulating with carpus

1 - metacarpal I articulates with carpus, metacarpals II and III reduced

2 - not articulating with carpus

134. Manual digit IV first phalanx, proportional length relative to metacarpal IV (ph1d4/mcIV) (modified from Kellner[56]: character 58):

0 - both small and reduced
1 - both enlarged with ph1d4 over four times the length of mcIV (ph1d4/mcIV>4.0)

2 - both enlarged with ph1d4 between four and two times the length of mcIV (4.0>ph1d4/mcIV>2.0)

3 - both enlarged with ph1d4 about or less than two times the length of mcIV (2.0≥ph1d4/mcIV>1.0)

4 - both enlarged with ph1d4 about the same or smaller than the length of mcIV (ph1d4/mcIV≤1.0)

135. Manual digit IV first phalanx, proportional length relative to tibiotarsus (ph1d4/ti) (modified from Kellner[56]: character 59):

0 - ph1d4 reduced

1 - ph1d4 elongated and less than twice the length of ti (ph1d4/ti <2.00)

2 - ph1d4 elongated about or longer than twice the length of ti (ph1d4/ti ≥2.00)

136. Manual digit IV second phalanx, proportional length relative to first phalanx (ph2d4/ph1d4) (modified from Kellner[56]: character 60):

0 - both short or absent

1 - elongated with second phalanx about the same size or longer than first (ph2d4/ph1d4 larger than 1.00)

2 - elongated with second phalanx up to 30% shorter than first (ph2d4/ph1d4 between 0.70 - 1.00)

3 - elongated with second phalanx more than 30% shorter than first (ph2d4/ph1d4 smaller than 0.70)

137. Manual digit IV third phalanx, proportional length relative to first phalanx (ph3d4/ph1d4) (Kellner[56]: character 61):

0 - both short or absent

1 - ph3d4 about the same length or larger than ph1d4

2 - ph3d4 shorter than ph1d4

138. Manual digit IV third phalanx, proportional length relative to the second phalanx (ph3d4/ph2d4) (Kellner[56]: character 62):

0 - both short or absent

1 - ph3d4 about the same size or longer than ph2d4

2 - ph3d4 shorter than ph2d4

139. Manual digit IV forth phalanx, proportional length relative to the first phalanx of manual digit IV (ph4d4/ph1d4):

0 - both short or absent

1 - both elongated, with the forth phalanx longer than the first (ph4/d4/ph1d4>1.00)

2 - both elongated with the forth phalanx the same length or shorter, but longer than 35% the length of the first (1.00>ph4d4/ph1d4>0.35)

3 - both elongated with the forth phalanx less than 35% the length of the first (ph4d4/ph1d4<0.35)

HIND LIMB

140. Femur, length relative to metacarpal IV length (fe/mcIV) (modified after Kellner[56]: character 63) (Andres & Ji[58]: char 110):

0 - femur about twice or longer than metacarpal IV (fe/mcIV ≥ 2.00)
1 - femur longer but less than twice the length of metacarpal IV (1.00 < fe/mcIV < 2.00)
2 - femur about the same length or shorter than metacarpal IV (fe/mcIV ≤ 1.00)

141. Metatarsal III, proportional length relative to tibia length (Kellner[56]: character 64):

0 - more than 30% of tibia length

1 - less than 30% of tibia length

142. Pedal digit V, number of phalanges (Kellner[56]: character 65):

0 - with four phalanges
1 - with 2 phalanges
2 - with 1 or no phalanx (extremely reduced)

143. Pes, second phalanx of digit V, shape (modified from Kellner[56]: character 66):

0 - reduced or absent
1 - elongated, straight
2 - elongated, curved
3 - elongated, very curved (boomerang shape)

EGG STRUCTURE

144. Eggs, external eggshell layer:

0 - hard shelled

1 - leathery, lacking calcium carbontate layer

2 - pliable, with thin calcium carbonate layer

Data matrix

Data Matrix in TNT File Format. Instructions: copy and paste the text below in a text editor such as Wordpad, save as a .tnt file and run on TNT.

xread

‘Holgado et al. On a new crested pterodactyloid from the Early Cretaceous of the Iberian Peninsula and the radiation of the clade Anhangueria. Scientific Reports’

144 57

Ornithosuchus_woodwardi
0000002000001000000000000000-0000--00--00100---000000000000--0-
0000000000?000000--
0000000000000000000000000000000000000000000000000000000000000?

Herrerasaurus_ischigualastensis
0000100000000000000000000000-0000--00--00100---000000000000--0-
00000000000000000--
0000000000000000000000000000000000000000000000000000000000020?

Scleromochlus_taylori
0000100000000000000002000000-0000--00--00100---00001?020??0--0-
00????00000?00000--
0000000?00?0000???00??0000??0?000???00??0???00??00?00000000???

Anurognathus_ammoni

00000010000000000010012000000-0100--00--00110---000110010??0--0-
00000000010000000--
000000010100000???0???01000?0???0??0110?1000000?10011222?0011?

Rhamphorhynchus_muensteri

1100100000011001110000120000-0000--00--00100---000000021000--0-
000000000100122010--
00000001000010000000000001000001000111002000110?1001222221012?

Darwinopterus_linglongtaensis

1011000000011??????000100000-000?200000000-1010100010021?00--0-
00?00?00???1?0000--
000000012000000???10??001?0?00?000?1110?100?310?10031111110221

Darwinopterus_robustodens

101100?00?011??????000100000-000?200000000-1010?00010021?00--0-
00?00?00???1?0000--
000000012000000???1???001?0?00??0??111??100?110?1003111111102?1

Pterodactylus_antiquus

1010000000011??0???000100000-0000--00--000-1001000010031?00--0-
10?00000???120000--
0000000120000000002110010000001000121100500?210?1003122222020?

Nyctosaurus_gracilis

1010100000011010010001220000-0000--00--000-?????00011021100--
1200?00000101132000--130-00010000--
011?110111000?00100013110130?0311221232222?20???

Muzquizopteryx_coahuilensis

?01010?000011???????0?????????00???????000-?????0001?021?00--
1200?????0????????0???30-00010000--
????11011????1????????11??30???11?21?????????1???

Pteranodon_longiceps
1010002000011160231001220000-0000--00--000-
112?000011023101231100100000101132000--030-00010000--
01111101110111012000121101411131122023222232020?

Tethydraco_regalis
??
??014111?????????????????

Dsungaripterus_weii
1010000101111041020000120000-1001110000010-?---
000010021101221101110100101122000--
11100011000000001111100110?000???0?2210??5?003101??131222?2120?

Noripterus_parvus
1010000101111??????000120000-1001110000010-?---
000010021?01221101?10?00?0?122000--
?110001100000000?11??0?????????????????????0??01??????????????

Tupuxuara_leonardii
1011002002031051021000120010-0001340000000-
?????0011002210127110110030010113200113130-00010000--
011111001???00002001?2100152013101?01313???2????

Thalassodromeus_sethi
1011002002031?61021000120010-0001340000000-
11100001100221012711011003001011?200113130-00010000--
0??

Caupedactylus_ybaka

1011002002031?51021002121010-0001340000000-
?????00?210221?12711011?000010113200110130-00010000--
0???????????00002001????015200?????????????????

Aymberedactylus_cearensis
???????????????????00??21??1220
011?130-0?010000--0??

Tupandactylus_imperator
1011002002031??????102121?10-0001331000000-
??????0110?22?01221101?????0???12210112?30-00010000--
0???

Caiuajara_dobruski
1011002002031??????102121110-0001331000?00-
???????1?00220?1221101101010??112210112130-00010000--
0?1111000?0?00002?01?21?11520?31?120????????????

Europejara_olcadesorum
??????2???031???????0???????????????????0??????????????220?12???0???????1?112?
10112?30-00010000--0???

Tapejara_wellnhoferi
10110020020310 2????102121110-0001330000000-
?????101100220012211011010101011221 0112130-00010000--
0?1111000?0?00002001?21011520031012 0?31????2120?

Sinopterus_dongi
1011002002031??????102121100-0001330000100-
1100010110022?11211101?0??10???12210110130-00010000--
0?1?11000???0000200??211?152??310?20?31222321???

Huaxiapterus_corollatus

101????????????????102121?00-0001330100100-
??????????0???????????????10???1?210110?30-00010000--
0?1?11??0???000???0??211??5???3??????31322321???

Huaxiapterus_benxiensis
1011002002031??????102121?00-0001330100100-
???????110022??1211101????10???12210110?30-00010000--
0?1?11??0????????????2????????3???20?31222?2120?

Eopteranodon_lii
101??0???2?????????102121100-00013300??100-
???????????????????????????????1?2001???30-00010000--
0????????????????????21???5???2??????3?222?2????

Chaoyangopterus_zhangi
101??1?0???????????001120?00-00????00--?00-
???????????????1??????????00???1?2000--?30-00010000--
0?102110????000???10?200??????310?20?31322?21?0?

Shenzhoupterus_chaoyangensis
1010012002031??????001120?00-0011550000000-
?????0??2????2?01241101????00???122000--?30-00010000--
0?1021??????0?0???1??200??5???310????31222321???

Jidapterus_edentus
101????????????????001120000-001???00--?00-
???????????????????????????0???132000--130-00010000--
0?1?2110??????0???1??200??5?0?310????3122232120?

Quetzalcoatlus_sp.
10100020020????????000120000-00014?00--?00-0---
000010??210?????????000010?132000--130-00010000--
011031221?0?00002010?20?0152003101???41322?2?20?

Zhejiangopterus_linhaiensis

10100020020?1??????000120000-0000--00--000-0---0000100?2?00--0-
01??0?00???1?2000--?30-00010000--
01??31221?0?000???10?200??5???310?20?413???2????

Azhdarcho_lancicollis

???????????????????00??2?00??0?????0?--??0-
?????????????1??????????0??0????????????30-00010000--
011031221??????????????015?0??????????????????

Hongshanopterus_lacustris

?????????????03011000?1100?0-0?????0?--
?0????????????????2??????00??0100101?????????000000100100001?11101??????????
??????????????????????????????

Nurhachius_ignaciobritoi

101100?0?00????????000110000-0000--00--000-?????01111?23?00--
??????0100?0?111000--
?20000010010000??111011???1?10200?2211?041??2212??1312???21???

Istiodactylus_latidens

1011000111111??????000100000-0000--00--000-11100011211230?0--0-
001?0?00?01111000--
?20000010011000?111?011???111020002?1?10411??212??????????????

Istiodactylus_sinensis

1011000111111??????000100000-0000--00--000-?????011211230?0--0-
00?00?00???11?000--
?200000100110001??11??1???1?1???0??21???4???221???131222??1???

Haopterus_gracilis

1010???????????????000110000-0000-?0?--?00-
?????0??????????????????????0??1121?00--
000000011000000???11??0???11111?0?22???04???211????3?222???20?

Ikrandraco_avatar

10100000000110????1000100000-0000--00--000-11001?01000311?0--0-00?001001011200011 2?000000100000001111101?????????????2????41??2112201??2????????

Lonchodraco_giganteus

1??????????????????00??00000-0?????0000???????????????????????????01?0???1?0?01?0?0?000012000001???

Tropeognathus_mesembrinus

1010002000011010011 00?1000010000102000000 0-?????01001021101001000100202101110001010001?0010000110?1111011????11???002???????10?21???????????????

AMNH_22555

101010200001101001100110000 2?0001020000000-11001010110211010010001001 02101???001?1000111010000110111110 1?1??21112100????104110?212??1???????????

Anhanguera_blittersdorffi

1010102000011010011001100002000010200000 00-?????01011021101001000100102101????0101?0011101000011 0??

Anhanguera_piscator

1010102000011?1????0011000020000102000000 0-1100101011021101001000100?0210112000101000111010000110111110 1?1012111210022111041102212??1??????2120?

Liaoningopterus_gui

101?0?2000011???????0??00002000?1020000???????????0110211??????????0102?????0??10100011101000011011 1?1???

Siroccopteryx_moroccensis

1???????????????????0??3000120??1??0000????????????????????????????01?2??????

??????0??10010000??0??

Uktenadactylus_wadleighi

1???????????????????0??3000120?????0000????????????????????????????01?2??????

??????0??100??00???0??

Coloborhynchus_clavirostris

1???????????????????0??3000120?????0000????????????????????????????01?2??????

??????0??100??00???0??

Ludodactylus_sibbicki

10100000000110?????00110000??000???0000000-

1100101001021101161100?00102??112000101?00111010000110??????????????????

????????????????????????????

Guidraco_venator

10?0000000011??????00110000??000???0000000-

?????0?0010211?1161100?00?02?0?12000?--

?00111010000110?1111??

Caulkicephalus_trimicrodon

10????0??0??????????01?000?2?0??10200000??????????????????11611????01?2?0???

???????00?110???0???

Cearadactylus_atrox

10100??0010?101????0?11000?2000?1020000??????00??1011?211??????????010210?

120000--00011101000011??

Maaradactylus_kellneri

101?0?2001011???????0??00002000?1020000??0?1??????0110211?1????????0102???

??0??10100011101000011??

Camposipterus_nasutus
1???????????????????0??0000100?????0000????????????????????????????01?1??????
??????0?0000??0???

Cimoliopterus_cuvieri
1???????????????????0??00000-
0??1?20000????????????????????????????01?1??????????0?0000??00???0?????????
??????????????????????????????????????

Ornithocheirus_simus
10??????????????????0??000?0-
0?????????????????????????????????????0??0????????????0?00001000011??????????
??????????????????????????????????????

Hamipterus_tianshanensis
10100020000?101101100010000100001210011000-?????001010211?0--0-
00100101101120000--
1001100100001101111101????1?????00????10410022?2?0???????????2

*Iberodactylus_andreui*_gen._et_sp._nov.
????????????????????0??000?100??1?10011????????????????????????????01?1??????
??????0??1001000011???
;

Wingspan estimation

In order to estimate the wingspan of *Iberodactylus andreui*, we present here wingspan estimations for individuals of *Hamipterus tianshanensis*. In the single block IVPP 18931, the following elements were found in association: two small skulls (IVPP 18931.1, and .2), a large skull (IVPP 18931.3), a scapula, a larger scapula, and a partial wing composed of ulna, metacarpal IV and the first wing phalanx. Based on the

proportions between the scapula and the other elements as seen in *Anhanguera piscator*, *Anhanguera* sp. (AMNH 22555) and "*Santanadactylus pricei*" (AMNH 22552) (Table S3). As the size of the larger scapula is proportional to the partial wing they most likely belongs together, and not the smaller scapula. The same is true for the large skull, based on the proportions between the skull and wing elements in *Anhanguera piscator* and AMNH 22555. We thus tentatively associate here the large skull (IVPP 18931.3) to the partial wing.

Estimates for the total wingspan of IVPP 18931.3 are given in Table S4 based on correlations to the wingspan and measures of each element in *Anhanguera piscator*, AMNH 22555 and AMNH 22552. In this way, based on Tables S2–4, we can conclude that a *Hamipterus tianshanensis* individual with a distance between the first and sixth pairs of alveoli of 96.9 cm probably had a wingspan between 3.05 and 3.29 meters. Scaling this wingspan range to the distance of 119 cm between the first and sixth pairs of alveoli seen in *Iberodactylus andreui* results in 3.75 and 4.04.

Table S2. Measurements of all the elements found in IVPP 18931. Sca: scapula; ul: ulna; mcIV: metacarpal IV; ph1d4: first wing phalanx.

	Jaw length	Distance 1st – 8th tooth	Sca (small)	Sca (large)	Ul	McIV	Ph1D4
Partial wing	-	-	-	73.46	248.18	186.36	339.59
Sca (small)	-	-	51.82	-	-	-	-
Skull 1	243	75	-	-	-	-	-
Skull 2	?224.85	69.4	-	-	-	-	-
Skull 3	?313.95	96.9	-	-	-	-	-

Table S3. Measurements of some wing elements found in *Anhanguera piscator* and *Anhanguera* sp. Sca: scapula; ul: ulna; mcIV: metacarpal IV; ph1d4: first wing phalanx.

	Jaw length	Sca	Ul	McIV	Ph1D4	Wingspan	Reference
Anhanguera piscator	533	112	390	256	?	4690	Kellner & Tomida, 2000
Anhanguera sp. (AMNH 22555)	396	?	291	206	446	3800	Wellnhofer, 1987
AMNH 22552	?	?	242.5	172.06	371.99	3270	Kellner et al., 2013

Table S4. Maximized wingspan of IVPP 18931.3 as estimated based on skull and wing elements of *Anhanguera piscator*, *Anhanguera* sp. (AMNH 22555) and AMNH 22552. MaxWs: maximized wingspan.

	MaxWs based on Skull	MaxWs based on Sca	MaxWs based on Ul	MaxWs based on McIV	MaxWs based on Ph1D4	Mean
Based on *Anhanguera piscator*	2762	3076	2985	3414	-	3059
Based on *Anhanguera* sp.	3012	-	3240	3437	2893	3145
Based on AMNH 22552	-	-	3346	3541	2985	3290

Supplementary references

48. Young, C. C. On a new pterosaurian from Sinkiang, China. *Vertebrate Palasiatica* **8**, 221–225 (1964).

49. Dalla Vecchia, F. M., Wild, R., Hopf, H. & Reitner, J. A crested rhamphorhynchid pterosaur from the Late Triassic of Austria. *J. Vert. Paleontol.* **22**(1), 196–199 (2002).

50. Stecher, R. A new Triassic pterosaur from Switzerland (Central Austroalpine; Grisons), *Raeticodactylus filisurensis* gen. et sp. nov. *Swiss J. Geosci.* **101**, 185–201 (2008).

51. Hieronymus, T. L. & Witmer, L. M. Homology and evolution of avian compound rhamphothecae. *The Auk* **127**(3), 590–604 (2010).

52. Roberts, E. M., Rogers, R. R. & Foreman, B. Z. Continental insect borings in Dinosaur bone: examples from the Late Cretaceous of Madagascar and Utah. *J. Paleontol.* **81**(1): 201–208 (2007).

53. Bader, K. S., Hasiotis, S. T. & Martin, L. D. Application of forensic science techniques to trace fossils on dinosaur bones from a quarry in the Upper Jurassic Morrison Formation, Northeastern Wyoming. *Palaios* **24**, 140–158 (2009).

54. Huchet, J. B. *et al.* Identification of dermestid pupal chambers on Southern Levant human bones: inference for reconstruction of Middle Bronze Age mortuary practices. *J. Archaeol. Sci.* **40**, 3793–3803 (2013).

55. Marin-Monfort, M. D., Holgado, B., García-Martínez, D., Bastir, M. & Suñer, M. First European evidences of pupal chambers on dinosaur bones from the Jurassic-Cretaceous boundary (Valencia, Spain). In: *New perspectives on the Evolution of*

Phanerozoic Biotas and Ecosystems (eds. Manzanares, E. et al.) 67 (Ayuntamiento de Alpuente, 2016).

56. Kellner, A. W. A. A new hypothesis of pterosaur phylogeny. I e II Simpósios sobre a Bacia do Araripe e bacias interiores do Nordeste, Comunicações. Crato, 249–258 (2001).

57. Unwin, D. M. Preliminary results of a phylogenetic analysis of the Pterosauria (Diapsida: Archosauria). Sixth Symposium on Mesozoic Terrestrial Ecosystems and Biota, 69–72 (1995).

58. Kellner, A. W. A. New information on the Tapejaridae (Pterosauria, Pterodactyloidea) and discussion of the relationships of this clade. *Ameghiniana* **41**(4), 521–534 (2004).

59. Andres, B. & Ji, Q. A new pterosaur from the Liaoning province of China, the phylogeny of the Pterodactyloidea, and convergence in their cervical vertebrae. *Palaeontology* **51**(2), 453–469 (2008).

60. Andres, B. & Myers, T. S. Lone Star Pterosaurs. *Earth Env. Sci. T. R. So.* **103**(3-4), 383–398 (2013).

61. Kellner, A. W. A. & Langston, W. Cranial remains of *Quetzalcoatlus* (Pterosauria, Azhdarchidae) from Late Cretaceous sediments of Big Bend National Park, Texas. *J. Vert. Paleontol.* **16**(2), 222–231 (1996).

62. Lü, J., Azuma, Y., Dong, Z., Barsbold, R., Kobayashi, Y. & Lee, Y. New material of dsungaripterid pterosaurs (Pterosauria: Pterodactyloidea) from western Mongolia and its palaeoecological implications. *Geol. Mag.* **146**(5), 690–700 (2009).

63. Martill, D. M. & Naish, D. Cranial crest development in the azhdarchoid pterosaur *Tupuxuara*, with a review of the genus and tapejarid monophyly. *Palaeontology* **49**(4), 925–941 (2006).

64. Pinheiro, F. L. Fortier, D. C., Schultz, C. L., Andrade, J. A. F. G. & Bantim, R. A. New information on the pterosaur *Tupandactylus imperator,* with comments on the relationships of Tapejaridae. *Acta Palaeontol. Pol.* **56**(3), 567–580 (2011).

65. Unwin, D. M. On the phylogeny and evolutionary history of pterosaurs. *Geol. Soc. London Spec. Publ.* **217**, 139–190 (2003).

66. Bennett, S. C. Taxonomy and systematics of the Late Cretaceous pterosaur *Pteranodon* (Pterosauria, Pterodactyloidea). *Occas. pap. Mus. Nat. Hist*. 1–70 (1994).

67. Wang, X., Kellner, A. W. A., Zhou, Z. & Campos, D. A. Pterosaur diversity and faunal turnover in Cretaceous terrestrial ecosystems in China. *Nature* **437**, 875–879 (2005).

68. Pêgas, R. V., Leal, M. E. & Kellner, A. W. A. A basal tapejarine (Pterosauria; Pterodactyloidea; Tapejaridae) from the Crato Formation, Early Cretaceous of Brazil. *PLoS One* **11**(9), e0162692; 10.1371/journal.pone.0162692 (2016).

69. Longrich, N. R., Martill, D. M. & Andres, B. Late Maastrichtian pterosaurs from North Africa and mass extinction of Pterosauria at the Cretaceous-Paleogene boundary. *PLoS Biology* **16**(4): e1002627; 10.1371/journal.pbio.2001663 (2018).
